# Novel Insights and Therapeutics in Multiple Sclerosis

**DOI:** 10.12688/f1000research.6378.1

**Published:** 2015-08-07

**Authors:** Catriona A. Wagner, Joan M. Goverman

**Affiliations:** 1 Department of Immunology, University of Washigton, Seattle, WA, 98109-8509, USA

**Keywords:** multiple sclerosis, immune, therapeutics

## Abstract

The last twelve years have witnessed the development of new therapies for relapsing-remitting multiple sclerosis that demonstrate increased efficacy relative to previous therapies. Many of these new drugs target the inflammatory phase of disease by manipulating different aspects of the immune system. While these new treatments are promising, the development of therapies for patients with progressive multiple sclerosis remains a significant challenge. We discuss the distinct mechanisms that may contribute to these two types of multiple sclerosis and the implications of these differences in the development of new therapeutic targets for this debilitating disease.

## Introduction

Multiple sclerosis (MS) is an inflammatory, demyelinating disease of the central nervous system (CNS) and is the most common cause of non-traumatic neurologic disability in young adults. Although the etiology of MS has been debated in the past, recent data from GWAS studies provide exceptionally strong evidence that MS is an autoimmune disease ^1– 3^. This view was originally based on the observation that experimental autoimmune encephalomyelitis (EAE), an animal model that recapitulates many important features of MS, is induced by the activation or adoptive transfer of self-reactive CD4+ T cells specific for myelin proteins ^4^. Furthermore, susceptibility to MS is most strongly associated with MHC class II alleles ^1^. Inflammatory lesions and plaques of demyelination in the CNS are considered hallmark features of MS; however, substantial heterogeneity (with respect to clinical course, pathology, and response to therapies) is seen among patients. This review will focus on current ideas regarding the role of different pathological mechanisms in shaping these different manifestations of MS. Achieving an understanding of the specific pathogenic pathways that are relevant to individual patients with MS is critically important in order to predict which patients will respond well to current therapies, and to identify new therapeutic targets that can be tailored for patients with different types of MS.

## Heterogeneity in MS

For the majority of patients with MS, disease course begins with a relapsing-remitting phase with intermittent, discrete periods of neurological symptoms that coincide with the appearance of inflammatory lesions. Over time, most patients with relapsing-remitting MS convert to a progressive stage called secondary progressive MS, characterized by a decrease in the frequency, or complete cessation, of relapses and gadolinium-enhancing MRI lesions, and the gradual accumulation of disability associated with brain and spinal cord atrophy. A small subset of patients with MS initially present with a progressive disease course that is not preceded by clinical exacerbations. This form of MS is called primary progressive MS because it is not preceded by a relapsing-remitting phase ^5, 6^.

Significant heterogeneity is also observed in the structure of lesions and types of tissue injury seen in patients with MS. Lesions have been grouped into four different patterns, based on the presence or absence of antibody deposition and complement activation, differential loss of certain myelin proteins, whether lesions occur at perivenous sites, and whether oligodendrocytes are spared or die by apoptosis or necrotic cell death ^7, 8^. The observation that individual patients with MS exhibit only one pattern of lesions led to the proposal that different patterns may arise from distinct pathogenic pathways. However, these patterns have not been associated with particular clinical disease courses. Differences are also seen among patients in the distribution of lesions between the brain and spinal cord. The vast majority of patients with relapsing-remitting MS exhibit numerous lesions in the cerebral white matter. However, a small subset (10–15%) of patients with MS exhibit lesions predominantly in the spinal cord with relatively sparse brain involvement.

## Relapsing-remitting MS pathogenesis and therapies

Relapsing-remitting MS is the best studied form of MS, as the majority of patients initially exhibit this form of disease, and the EAE model recapitulates many aspects of the inflammatory lesions seen in relapsing-remitting MS. Both MRI and immunohistochemical analyses of tissue sections indicate that inflammation is the key component leading to tissue injury and clinical relapses in relapsing-remitting MS patients. Perivascular lesions are comprised of inflammatory infiltrates dominated by lymphocytes and myeloid cells. Our conceptual framework for understanding how these lesions arise is based on studies in EAE. In EAE, activation of myelin-specific T cells induces expression of adhesion molecules and integrins that facilitate their extravasation across the blood brain barrier (BBB). Upon entry into the CNS, myelin-specific T cells are reactivated by the small number of antigen-presenting cells (APCs) in the healthy CNS that constitutively present myelin antigens in the perivascular and subarachnoid spaces. This reactivation triggers the T cells to produce soluble, inflammatory mediators that cause BBB permeability and recruitment of a range of inflammatory leukocytes ^4^. Formation of a localized, inflammatory environment within lesions results in plaques of demyelination and axonal damage.

In EAE, CD4+ T cells are the predominant lymphocyte in the infiltrate, as the protocol for inducing EAE specifically primes CD4+ T cells. However, there is an abundance of CD8+ T cells in lesions in MS patients, and clonal expansion indicative of antigen-driven activation is more evident in the CD8+ T cell subset in the blood and cerebrospinal fluid of MS patients. Furthermore, depletion of CD4+ T cells resulted in limited therapeutic efficacy in MS patients ^9^, although the results of this trial may not be conclusive. In contrast, treatments that deplete all leukocytes demonstrated greater efficacy in MS patients ^10^. These observations raise key questions ( Box 1) about the role for different lymphocyte subsets in CNS autoimmunity.

Box 1. **Outstanding questions regarding the role of lymphocyte subsets in relapsing-remitting MS**

- Do both CD4 and CD8 T cells contribute to the pathogenesis of MS? What are their relevant effector mechanisms?
- Do CD8+ T cells exhibit both regulatory and pathogenic activity as seen for different subsets of CD4+ T cells?
- How do B cells contribute to the pathogenesis of MS?

### The case for CD4 T cells

An important role for CD4+ T cells in MS is clear from the strong association of MHC class II molecules with disease susceptibility ^1^. Studies of effector CD4+ T cells in EAE implicated both IFN-γ-producing Th1 and IL-17-producing Th17 cells as pathogenic mediators ^11^. Originally, Th1 cells were considered the main effector cells, as adoptive transfer of Th1 cells could induce EAE ^12, 13^. This finding was consistent with an earlier observation that MS was exacerbated by the administration of IFN-γ ^14^. However, mice deficient in cytokines associated with the differentiation and function of Th1 cells developed severe EAE ^15^. In contrast, deficiency in IL-23, which is important for Th17 cell stabilization, conferred resistance to EAE, suggesting that Th17 cells may be the true effector cells ^16^. An increase in IL-17 transcripts was also reported in chronic MS lesions ^17^. However, IL-17A-/- and IL-17F-/- mice, treated with anti-IL-17A blocking antibody, are still susceptible to EAE ^18^. Subsequently, GM-CSF was reported to be essential for EAE pathogenesis ^19– 21^, and recent studies have suggested that T cells producing GM-CSF may represent a distinct T cell subset ^22, 23^.

The studies described above were all carried out in C57BL/6 mice. Our studies of EAE in C3Heb/Fej mice have provided further insight into the complexity of EAE pathogenesis. While the incidence of EAE in C3Heb/Fej mice is strongly reduced in mice lacking both IL-17RA and IFN-γ receptors, disease incidence was only modestly reduced in mice that lacked either only IL-17 or only IFN-γ signaling. Importantly, we found that IFN-γ and IL-17 signaling had a much greater impact on the pattern of lesion localization within the CNS. IL-17 promoted inflammation in the brain *via* induction of chemokines that recruit neutrophils, and neutrophils contributed significantly to parenchymal tissue damage in the brain ^24^. In contrast, IFN-γ inhibited inflammation in the brain by inhibiting neutrophil recruitment. Surprisingly, IFN-γ exerted the opposite influence in the spinal cord by promoting both neutrophil recruitment and inflammation in this microenvironment. Despite the enhanced, IFN-γ-mediated neutrophil recruitment to the spinal cord, neutrophils contributed less to spinal cord tissue damage compared to their role in the brain ^24^. Recent work by Segal and colleagues in C57BL/6 mice also suggested that neutrophils may be more important effector cells in the brain compared to the spinal cord ^25^. Interestingly, in contrast to EAE in C57BL/6 mice, we have observed only a modest reduction in disease incidence in C3Heb/Fej mice when EAE is induced by adoptive transfer of GM-CSF-/- T cells in C3Heb/Fej mice, suggesting that the stringent requirement for GM-CSF seen in C57BL/6 mice may be a strain-specific finding (Pierson E.R., Johnson M.C., and Goverman J.M., unpublished observations). Collectively, these studies demonstrate that it is critical to study different mouse strains in order to understand the complexity of disease manifestation in MS patients.

The finding that the brain and spinal cord microenvironments in mice respond very differently to cytokines produced by infiltrating T cells suggests that patients with distinct neuroinflammatory patterns may respond quite differently to therapies that target specific cytokines. There are also other challenges in designing cytokine-based therapies for MS patients. Despite the substantial data supporting key roles for Th1 and Th17 cells in EAE, a clinical trial administering ustekinumab (an antibody that neutralizes cytokines that promote differentiation of both Th1 and Th17 cells), had no beneficial effect ^26^. It is difficult to draw conclusions from this one trial, however, especially in light of the fact that it is not known whether effector T cell differentiation occurs in the periphery or the CNS, or how important ongoing T cell differentiation is in patients with established MS. The dramatic benefit seen in patients with psoriasis following administering of an IL-17-neutralizing antibody has also not yet been reported for similar clinical trials in patients with MS. It is possible that better stratification of patients with MS, with respect to their neuroinflammatory pattern and other key disease characteristics, is needed to properly evaluate the effectiveness of therapeutic targeting of specific cytokines.

### The case for CD8 T cells

CD8+ T cells often predominate in tissue sections and in CSF of MS patients, and clonal expansion is more commonly observed in the CD8+ compared to the CD4+ T cell subset ^4, 27, 28^. However, the role of CD8+ T cells is still unclear, as EAE models have pointed to both pathogenic and regulatory functions. Global elimination of CD8+ T cells using either CD8-/- mice or antibody-mediated depletion of CD8+ T cells *in vivo* suggested a regulatory role for CD8+ T cells ^29, 30^. The observations that Qa-1-deficient mice exhibit increased susceptibility to EAE, and that adoptive transfer of Qa-1-restricted CD8+ T cells ameliorates disease, suggested that there may be distinct regulatory subsets of CD8+ T cells ^31, 32^. Other studies have reported a pathogenic role for myelin-specific CD8+ T cells in CNS autoimmunity ^33– 35^, and animal models using neo-antigens expressed in the CNS and CD8+ T cells that recognize the neo-antigen support a pathogenic role for CD8+ T cells ^36– 40^. We identified CD8+ T cells that recognize a MHC class I-restricted myelin basic protein (MBP) epitope and showed that these CD8+ T cells were pathogenic and produced lesions distinct from those seen in conventional EAE but similar to some lesions seen in patients with MS ^33, 41, 42^. We also showed that both dendritic cells and oligodendrocytes presented the MHC class I-restricted epitope of MBP within the CNS of mice with CD4+ T cell-initiated EAE ^43^. We speculate that CD8+ T cells could be pathogenic if they are triggered to produce inflammatory cytokines upon encountering dendritic cells and/or lyse oligodendrocytes, but they might ameliorate disease if they subsequently lyse dendritic cells that present antigen to both CD4+ and CD8+ T cells within the CNS. Our preliminary data suggest that recruitment of MBP-specific CD8+ T cells during disease induction can exacerbate CD4+ T cell-initiated EAE and may enhance brain inflammation (Wagner C.A. and Goverman J.M., unpublished data). However, CD8+ T cells may play different roles at different phases of disease, and it is important to identify their specific effects during each disease stage in order to therapeutically target (or harness) their activity.

### The case for B cells

A pathogenic role for B cells in MS is suggested by the therapeutic benefit observed in patients treated with anti-CD20 monoclonal antibodies (rituximab or ocrelizumab) that deplete B cells ^44, 45^. As anti-CD20 does not deplete antibody-producing plasma cells, the ability of B cells to present antigen to T cells may be critical in CNS autoimmunity. In animal models, B cells have been shown to promote EAE induction by acting as antigen-presenting cells for T cells in both the periphery ^46– 49^ and CNS ^50^. We found that in the healthy CNS, B cells comprise the majority of MHC class II+ cells, and that they play a role in the initial reactivation of infiltrating myelin-specific T cells, specifically the Th1 subset ^50^. Despite the therapeutic efficacy of B cell depletion in MS, B cells that produce IL-10 and ameliorate EAE have been described ^51, 52^. If these regulatory B cells retain a stable phenotype in patients, transfer of this subset may be beneficial, in addition to depleting B cells that function only to present antigen to T cells ^53^.

## Secondary progressive MS pathogenesis and therapies

Following the success of anti-inflammatory therapies in relapsing-remitting MS, clinical trials were initiated in secondary progressive MS cohorts. However, clinical trials analyzing the effects of beta-interferon found no significant treatment effect, particularly in patients that had not exhibited MRI lesions for several years ^54– 57^. The lack of efficacy of anti-inflammatory therapies in secondary progressive MS suggested that neurodegeneration proceeds independently of inflammation in this stage ^58^. However, further analyses of normal-appearing white matter and meninges tissue sections revealed that inflammation was still present, albeit in a different form ^59^. Staining, using a marker that selectively stains for leaky endothelial cells, indicated that inflammation is compartmentalized behind a less permeable BBB ^60^. Differences in the pathology, clinical signs and response to current therapies suggest that different mechanisms predominate in progressive MS, requiring new therapeutic approaches.

Secondary progressive MS is characterized by increasing brain atrophy and accumulation of irreversible axonal and neuronal degeneration. Gadolinium-enhancing MRI lesions subside ^61^ and a diffuse pattern of inflammation predominates in normal-appearing white and grey matter ^62^. The mechanisms underlying these changes in pathology are poorly understood. Axonal transection begins early in relapsing-remitting MS ^63^; however, the mechanisms may differ in these two stages of disease. During relapsing-remitting MS, inflammatory cells are thought to mediate demyelination *via* secretion of degradative enzymes, production of oxidative products, and increased levels of glutamate that can damage oligodendrocytes *via* excessive NMDA receptor signaling ^64^. Thus, transected axons are more abundant in active lesions where inflammatory cells are localized *versus* chronic lesions in relapsing-remitting MS ^63^. In contrast, demyelination and axonal injury is primarily associated with microglia activation in secondary progressive MS ^65^. The pattern of diffuse parenchymal inflammation, as well as the presence of T and B cells in the meninges, may contribute to microglial activation; however the role of adaptive immune cells in facilitating, *versus* responding to, microglial activation remains to be established. A major consequence of microglial activation is the production of reactive oxygen species. Mitochondria and mitochondrial DNA are very susceptible to oxidative injury ^66, 67^, and axons are extremely susceptible to mitochondrial dysfunction due to their high demand for ATP production to propagate action potentials ^68^. Failure of remyelination in progressive disease is another key difference between relapsing-remitting MS and secondary progressive MS. The state of chronic demyelination leads to diffusion of Na+ channels away from nodes of Ranvier and a subsequent influx of sodium and increased ATP consumption. The resulting energy imbalance ultimately leads to axonal degeneration and tissue damage ^64^. Thus, remyelination may be a critical therapeutic target in secondary progressive MS, together with strategies to dampen microglial activation. Potential contributions of adaptive immune cells may also need to be addressed in secondary progressive MS.

An additional challenge in designing effective therapies for secondary progressive MS is our lack of understanding of mechanisms that lead to subpial demyelination. Therapies that prevent or resolve subpial lesions may be very important in treating secondary progressive MS patients as increased progression in disability is associated with cortical atrophy ^69^, and subpial lesions are represented to a greater extent than leukocortical lesions in the total cortical lesion load ^62, 70^. However, subpial lesions typically lack peripheral inflammatory cells and the mechanism of demyelination that produces these lesions, while unknown, may be distinct from mechanisms of demyelination in white matter. Additionally, meningeal inflammatory aggregates are present in some patients with secondary progressive MS and some studies have identified lymph follicle-like structures. Post-mortem analyses of brain and spinal cord tissue revealed proliferating B cells and follicular dendritic cells in the follicles, suggesting germinal center formation ^71^. The presence of these follicles correlates with disease severity and the extent of demyelination ^72^. However, the contributions of these follicles to disease pathogenesis are still unclear.

## Concluding remarks

In this review, we have highlighted potential differences in pathogenic mechanisms between relapsing-remitting MS and secondary progressive MS, and how these differences may require distinct therapeutic approaches. Lesions in relapsing-remitting MS patients arise from a complex interplay of both CD4+ and CD8+ T cells, with B cells and innate immune cells playing critical roles in orchestrating T cell responses. The efficacy of therapies such as Natalizumab ^73^ and fingolimod ^74, 75^ that reduce T cell entry into the CNS highlight the key role played by T cells in relapsing-remitting MS. While current therapies are relatively effective at reducing new MRI lesion formation and relapse rates, they are broadly anti-inflammatory and often associated with side effects. New therapies capable of targeting inflammation relevant only to the CNS are needed. In addition, patients with relapsing-remitting MS exhibit heterogeneity in CNS lesions, including the distribution of lesions within the CNS, reinforcing the need for treatments tailored to the individual patient. Finally, the long-term impact of reducing inflammatory lesions and clinical relapses during relapsing-remitting MS on the progression of neurological disability remains to be firmly established.

The distinct pathology seen in secondary progressive MS patients suggests that targeting different disease mechanisms may be important. Specifically, therapies that promote remyelination and prevent microglia activation, mitochondria dysfunction, and oxidative damage, while beneficial in relapsing-remitting MS, appear particularly crucial in treating patients with secondary progressive MS. It is also important to determine the exact role of inflammation during this phase to prevent relapse after tissue damage has been resolved. Because the BBB is more intact in secondary progressive MS, developing therapeutic agents capable of crossing the BBB is an additional challenge. A key to developing therapies for secondary progressive MS is the generation of new animal models that better reproduce the key features seen in this disease. While we have focused on secondary progressive MS, similarities in the pathology seen between secondary progressive MS and primary progressive MS support the hope that the same therapies will be beneficial in both types of disease.
