# Supplementary material for: Novel Insights and Therapeutics in Multiple Sclerosis
Source: F1000Res. 2015 Aug 7;4(F1000 Faculty Rev):517. [Version 1] doi: 10.12688/f1000research.6378.1 (PMC4544373; doi:10.12688/f1000research.6378.1)
Supplement: Peer review history

# Peer review: Referee response for version 1

**DOI:** 10.5256/f1000research.6839.r9922

**Published:** 2015-08-07

**Authors:** Benjamin M. Segal, M.D.

**Affiliations:** 1 Neurology Department, University of Michigan, Ann Arbor, MI, 48109, USA

I have read this submission. I believe that I have an appropriate level of expertise to confirm that it is of an acceptable scientific standard.

# Peer review: Referee response for version 1

**DOI:** 10.5256/f1000research.6839.r9926

**Published:** 2015-08-07

**Authors:** Melissa Brown

**Affiliations:** 1 Department of Microbiology-Immunology, Northwestern University Feinberg School of Medicine, Chicago, IL, 60611, USA

I have read this submission. I believe that I have an appropriate level of expertise to confirm that it is of an acceptable scientific standard.

# Peer review: Referee response for version 1

**DOI:** 10.5256/f1000research.6839.r9920

**Published:** 2015-08-07

**Authors:** Lars Fugger

**Affiliations:** 1 Nuffield Department of Clinical Neurosciences, University of Oxford, Oxford, OX3 9DU, UK

I have read this submission. I believe that I have an appropriate level of expertise to confirm that it is of an acceptable scientific standard.
